# Preparation of TiO_2_/WO_3_/C/N Composite Nanofibers by Electrospinning Using Precursors Soluble in Water and Their Photocatalytic Activity in Visible Light

**DOI:** 10.3390/nano11020351

**Published:** 2021-02-01

**Authors:** Vincent Otieno Odhiambo, Chra Rasool M. Mustafa, Le Ba Thong, Zoltán Kónya, Csaba Cserháti, Zoltán Erdélyi, István Endre Lukác, Imre Miklós Szilágyi

**Affiliations:** 1 Department of Inorganic and Analytical Chemistry, Budapest University of Technology and Economics, Szent Gellért tér 4., H-1111 Budapest, Hungary; chra.rasool@yahoo.com (C.R.M.M.); kenty9x@gmail.com (L.B.T.); 2 Department of Applied and Environmental Chemistry, University of Szeged, Rerrich Béla tér 1., H-6720 Szeged, Hungary; konya@chem.u-szeged.hu; 3 Department of Solid-State Physics, Faculty of Sciences and Technology, University of Debrecen, Bem ter 18/b, H-4026 Debrecen, Hungary; cserhati.csaba@science.unideb.hu (C.C.); zoltan.erdelyi@science.unideb.hu (Z.E.); 4 Research Institute for Technical Physics and Materials Science, Hungarian Academy of Sciences, Konkoly Thege M. út 29-33., H-1121 Budapest, Hungary; lukacs.istvan@energia.mta.hu

**Keywords:** electrospinning, TiO_2_ precursor soluble in water, composite nanofibers, photocatalysis

## Abstract

Extending the absorption range of TiO_2_ nanofibers to visible light is a great improvement of the photocatalytic property of TiO_2_. In this study, TiO_2_/WO_3_/C/N nanofibers were prepared by electrospinning using precursors soluble in water then annealing in argon. Titanium(IV) bis(ammonium lactato)dihydroxide (TiBALDH) and ammonium metatungstate (AMT) were used as the precursor for TiO_2_ and WO_3_ respectively. Different volume ratios of the precursors were added to a solution of PVP before electrospinning. The fibers were studied by XPS, SEM-EDX, TEM, FTIR, XRD, Raman spectroscopy and UV–VIS diffuse reflectance spectroscopy (DRS). The photocatalytic degradation of methylene blue by the fibers in visible light was investigated. The fibers had anatase TiO_2_ and monoclinic WO_3_. Based on UV–VIS DRS and Kubelka-Munk function the fibers could absorb visible light. Moreover, 100% TiBALDH had an indirect band gap of 2.9 eV, and the band gap decreased with increase in AMT, i.e., for 0% TiBALDH, band gap was 2.4 eV. The fibers degraded methylene blue dye in visible light, and 90% TiBALDH had the highest photocatalytic activity, i.e., it degraded 40% of the dye after 240 min.

## 1. Introduction

Photocatalytic reactions are advanced reduction and oxidation process widely used in water and cleansing systems, self-cleaning of surfaces, hydrogen production, and photoelectrochemical reactions [1,2,3]. TiO_2,_ a heterogeneous photocatalyst, has been demonstrated to effectively degrade organic pollutants in the environment into products such as CO_2_ and H_2_O. However, it has a significant setback of high bandgap that makes it utilize only UV light during photocatalysis, also photogenerated electron–hole pairs recombine very fast [4,5,6,7]. Many studies have been done to prepare high-performance TiO_2_ catalysts by making nanostructure composites of TiO_2_ with noble metals and other semiconductor oxides like WO_3_ [8,9,10]. Coupling TiO_2_-based nanofibers with heteroatom dopants lead to extra energy levels in the TiO_2_ band gap that allows for the absorption of visible light photons [11]. TiO_2_/WO_3_ and TiO_2_/carbon composites have been widely reported to have a lower band gap and reduced charge recombination rate. This improves photocatalytic degradation efficiency and charge transfer characteristics of the catalyst [12,13]. Nitrogen is a preferred nonmetal dopant of TiO_2_; it has low ionization energy, its atomic size is similar to oxygen, and it substitutes oxygen in the base lattice [14].

In several studies, different synthesis methods are combined to prepare TiO_2_-based composite photocatalysts. While this approach tries to maximize each method’s advantages, it will increase the number of procedures spent preparing the composite photocatalyst [15,16,17]. The challenge of improving TiO_2_ photocatalytic activity in a cost-efficient way justifies the need for further studies in this field [18]. Electrospinning is a simple and adaptable procedure used to prepare long fibers with diameters in the range of tens to hundreds of nanometers for application in many fields [19,20,21,22].

Zhao et al. coupled self-assembly of polystyrene-block-poly(ethylene oxide)-containing titanium-tetraisopropoxide and tungsten hexaphenoxide with electrospinning technique to fabricate hierarchically porous TiO_2_/WO_3_ composite nanofibers [23]. Simsek et al. synthesized TiO_2_/WO_3_/carbon composite for enhanced photocatalytic performance by controlled carbonization of a cellulosic precursor and solvothermal synthesis [24]. Hu et al. prepared TiO_2_/WO_3_ nanofibers coated with carbon by combining the electrospinning process with hydrothermal synthesis for enhanced hydrogen catalytic production [25]. Choi et al. reported fabricating TiO_2_/WO_3_-based films doped with chlorine or nitrogen for self-cleaning glass applications by a sol–gel spin coating method using HCl and HNO_3_ [26]. Lee et al. successfully synthesized WO_3_–N–TiO_2_ nanosheets using a combined sonochemical impregnation procedure for the photocatalytic treatment of harmful organic vapor [27]. Gao et al. synthesized TiO_2_-N-*x*%WO_3_ nanoparticles by synthesizing nitrogen-doped TiO_2_ powder using hydrolysis of TiCl_4_ by ammonia and then introducing WO_3_ into them by forming suspension with tungstic acid; the suspension was dried and then thermally treated. The composite nanoparticles had a more significant photocatalytic property than TiO_2_ and nitrogen-doped TiO_2_ in UV and visible light [28]. There are no reports of preparation of TiO_2_/WO_3_/C/N composite nanofibers by electrospinning using precursors of TiO_2_ and WO_3_ that dissolve in water.

Previously, we used electrospinning and precursors soluble in water to prepare TiO_2_/WO_3_ composite nanofibers and demonstrated that conditions of heat treatment influenced the nanofibers’ final composition [29]. When the fibers are annealed in inert conditions, the polymer decomposes without undergoing combustion. The semiconductor oxides are formed within the resulting carbon matrix [30].

In this study, TiO_2_/WO_3_/C/N composite nanofibers were prepared by electrospinning and annealing in argon. Water-soluble titanium(IV) bis(ammonium lactato)dihydroxide (TiBALDH) was the precursor for TiO_2_, while ammonium metatungstate (AMT) was the source of WO_3_. C and N are in the amorphous char material, which is the polymer’s residue after annealing in argon. The fibers were studied by XPS, SEM-EDX, TEM, FTIR, X-ray diffraction, Raman spectroscopy, and diffuse reflectance UV–VIS spectroscopy. The rate of the fibers to catalytically degrade methylene blue in visible light was investigated.

## 2. Materials and Methods

### 2.1. Synthesis of N Doped TiO_2_/WO_3_/C Nanofibers

Chemicals were of analytical grade, acquired from Sigma Aldrich (Budapest, Hungary) and used as obtained. The fibers were prepared by electrospinning. Titanium(IV) bis(ammonium lactato)dihydroxide (TiBALDH) solution was added to an aqueous solution of (ammonium metatungstate) AMT in different volume ratios (100%, 90%, 50%, 10%, and 0%). Then, 2 mL of this mixture was added to 2 mL of a polymer solution made by adding 0.5 mg polyvinylpyrrolidone (PVP) in a solution of ethanol and acetic acid in equal volume ratio. The mixture was stirred for 8 h at room temperature before electrospinning using a voltage of 20 kV and an intrusion rate of 1 mLh^−1^. The distance of the collector from the needle tip was 12 cm. The electrospun fibers were thermally treated in argon at 60 °C per hour till 600 °C.

### 2.2. Characterization of the Nanofibers

The surface morphologies and composition of the nanofibers were investigated by X-ray photoelectron spectrometer (XPS) (Phoibos, Berlin, Germany), scanning electron microscope (SEM) (JEOL Ltd., Tokyo, Japan) coupled with energy-dispersive X-ray spectrometer (EDX) (JEOL Ltd., Tokyo, Japan), and transmission electron microscope (TEM) (JEOL Ltd, Tokyo, Japan). SPECS X-ray photoelectron spectrometer (Berlin, Germany) fitted with a dual anode X-ray source, XR-50, and a PHOIBOS 150 energy analyzer (Berlin, Germany) was used to obtain the XPS spectra. Powdered samples were pressed onto indium foil for mounting. For the measurements, Al Kα X-ray source was operated with 150 W (14 kV). Survey spectra were acquired using 1 eV step size and 40 eV of pass energy. High-resolution spectra were acquired with a step size of 0.1 eV and a pass energy of 20 eV. All high-resolution spectra were charge corrected considering the C-C peak of the C 1 s spectrum to be at 284.8 eV [31]. The number of scans varied between 10 and 25. SEM-EDX measurements were done using a JEOL JSM-5500LV scanning electron microscope (JEOL Ltd., Tokyo, Japan) in a high vacuum mode. TEM measurements of the annealed fibers were done by sonicating the fibers in ethanol for 10 min, and then the liquids containing the fibers dropped onto a C-coated TEM grid. JEOL 200 FX-II transmission electron microscope (JEOL Ltd., Tokyo, Japan) was used for producing bright-field images from the different fibers.

Attenuated total reflection Fourier-transform infrared spectroscopy (ATR-FTIR) measurements of electrospun and annealed nanofibers were done using a Bruker Tensor 37 with a Specac Golden Gate ATR accessory (Billerica, MA, USA).

X-ray diffraction patterns were collected by a PANalytical X’Pert Pro MPD X-ray diffractometer (PANalytical, Almelo, Nerthalands) using Cu K_α_ irradiation. Raman spectra were obtained using a Jobin Yvon LabRAM Raman spectrometer (Horiba, Kyoto, Japan) equipped with an Olympus BX41 microscope (Olympus, Tokyo, Japan. The radiation source was Nd-YAG laser (Tokyo, Japan) operating at 532 nm.

UV–VIS DRS of the fibers were measured by AvaSpec 2048 with a Fiber Optic Spectrophotometer (Avantes BV, Apeldoorn, Netherlands) between 250 and 800 nm. The fibers’ optical band gap energy was determined using absorption edge from UV–VIS DRS and Tauc plots.

### 2.3. Photocatalysis in Visible Light

For this, 1.0 mg of the nanofibers was added to a 3 mL aqueous solution of methylene blue dye with a concentration of 0.0126 g/L in a quartz cuvette. To obtain adsorption equilibrium, the samples were put overnight in a dark place. The photocatalytic degradation of methylene blue after exposure to visible light was investigated by recording the absorbance of the peak at 664 nm for every 30 min for 4 h using a Jasco V-550 UV–VIS spectrometer (Jasco, Tokyo, Japan).

## 3. Results and Discussion

**Figure 1:**
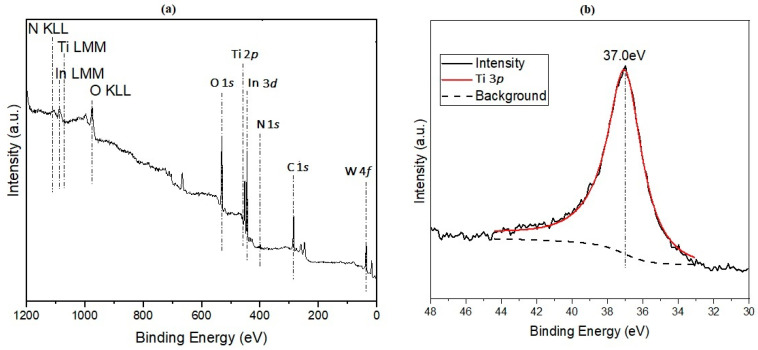
(**a**) XPS survey spectrum of 50% TiBALDH fibers and (**b**) Ti 3*p* XPS spectrum of 100% TiBALDH fibers.

**Figure 2:**
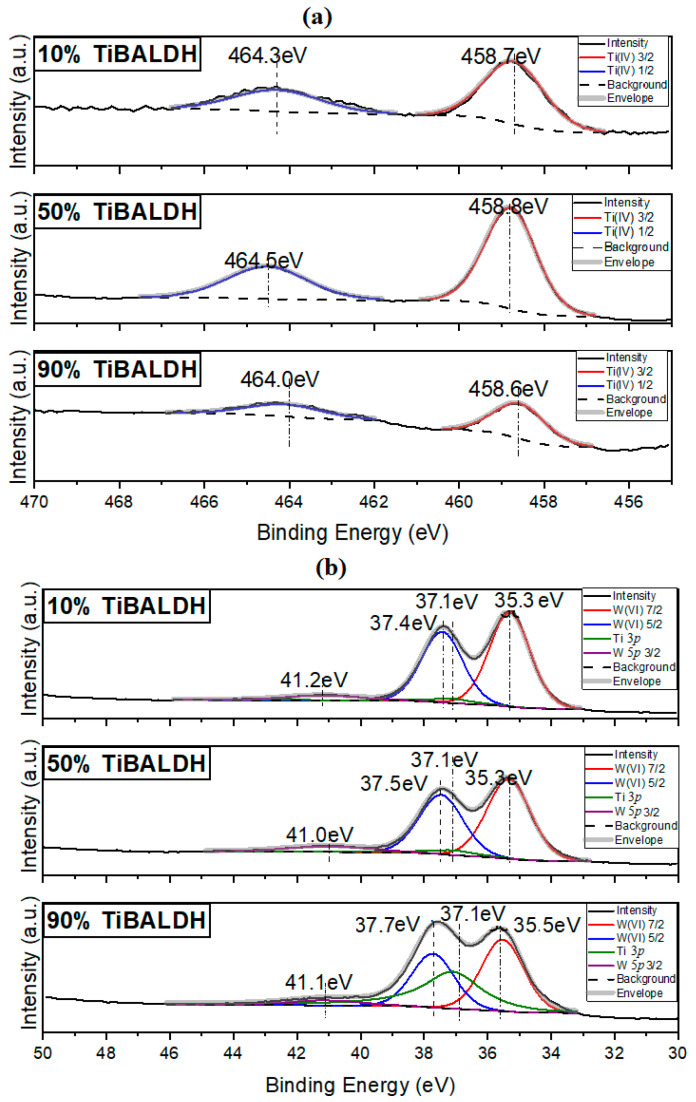
(**a**) Ti 2*p* XPS spectrum of composite fibers and (**b**) W 4*f* spectrum of composite fibers.

XPS spectra of the fibers after heat treatment in argon are presented in Figure 1 and Figure 2. Figure 1a is a survey spectrum of the 50% TiBALDH composite fibers. Based on the spectrum, the following elements were detected: C, O, Ti, W, and N; their main regions are marked on the survey spectra. The N 1*s* peak around 400 eV can be attributed to substitution of oxygen in the base lattice by nitrogen to form N-Ti-O [11]. Due to the use of In foil, In was also identified. Figure 1b shows the Ti 3*p* spectrum obtained from 100% TiBALDH fibers. Since the Ti 3*p* spectrum region overlaps the W 4*f* spectrum region, this Ti 3*p* peak shape was used to precisely distinguish between the two elements.

Figure 2a shows the Ti 2*p* XPS spectrum of the composite fibers. For all of the XPS signals, the Ti_2*P*1/2_ peak was observed at 464.3 eV, while the Ti_2P3/2_ peak was observed at 458.7 eV; this was consistent with Ti^4+^ [32,33]. According to Figure 2b, for all the composite fibers, W4*_f_*_7/2_ peaks had binding energy of 35.3 eV, while W4*_f_*_5/2_ peaks had 37.5 eV characteristic of W^+6^ coordinated by oxygen atoms [34]. The relative intensities of the elements were used to calculate the surface concentration of the various elements in the fibers. The result was further corroborated with SEM-EDX. Table 1 shows the elemental composition of the annealed fibers from XPS and EDX data. The Ti/W based on the atomic percent was different from the volume ratios of the precursors; 90% TiBALDH had Ti/W of 3.5, 50% TiBALDH had Ti/W of 0.31, and 10% TiBALDH had Ti/W of 0.22. This is because although TiBALDH contains only 16% titanium, AMT contains 30% tungsten. The high amount of carbon in the samples can be due to carbon formation within the fibers’ structure because of the decomposition of the as-spun fibers without combustion. Nitrogen is also in the carbon matrix formed when the as-spun fibers’ polymer part is pyrolyzed.

**Figure 3:**
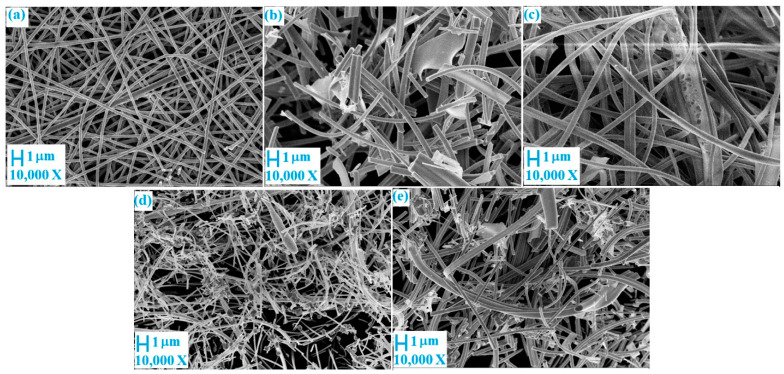
SEM photographs of fibers after annealing: (**a**) 100% TiBALDH, (**b**) 90% TiBALDH, (**c**) 50% TiBALDH, (**d**) 10% TiBALDH, and (**e**) 0% TiBALDH.

**Figure 4:**
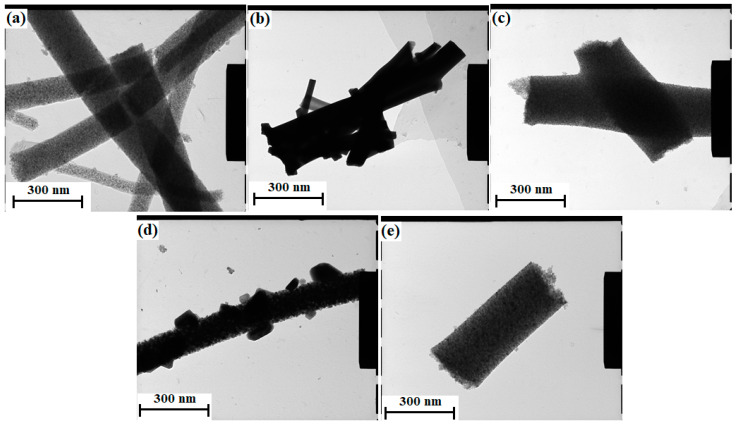
TEM photographs of fibers after annealing: (**a**) 100% TiBALDH, (**b**) 90% TiBALDH, (**c**) 50% TiBALDH, (**d**) 10% TiBALDH, and (**e**) 0% TiBALDH.

**Figure 5:**
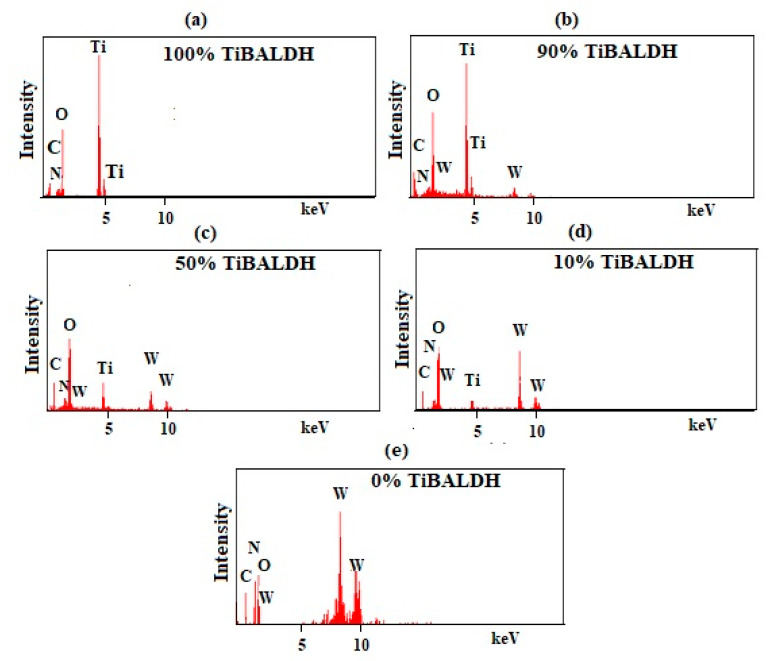
Energy-dispersive X-ray (EDX) spectra of the annealed fibers: (**a**) 100% TiBALDH, (**b**) 90% TiBALDH, (**c**) 50% TiBALDH, (**d**) 10% TiBALDH, and (**e**) 0% TiBALDH.

Figure 3 and Figure 4 are images of the samples after annealing obtained by SEM and TEM, respectively. The images show that the samples were fibrous. The diameter of the samples increased with a decrease in the amount of TiBALDH. Further, 100% TiBALDH fibers had a diameter of between 150 and 200 nm, while 0% TiBALDH fibers had a diameter of between 300 and 400 nm. The EDX spectra and elemental composition of the samples are shown in Figure 5. The spectra confirmed the presence of C, N, O, Ti, and W in the composite fibers.

**Figure 6:**
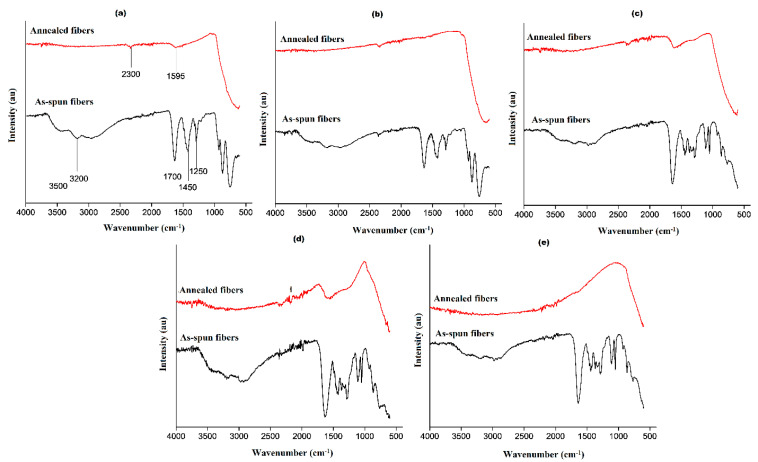
FTIR spectra of fibers before and after annealing: (**a**) 0% TiBALDH, (**b**) 10% TiBALDH, (**c**) 50% TiBALDH, (**d**) 90% TiBALDH, and (**e**) 100% TiBALDH.

Fourier-transform infrared spectra obtained before and after annealing the fibers are presented in Figure 6. The as-spun fibers have peaks between 3500 and 3200 cm^−1^ that can result from O-H stretching bonds from water and N-H stretching bonds from AMT [35]. The peak observed at about 2900 cm^−1^ is due to C-H vibrations from TiBALDH and PVP. Vibrations by C=O bonds were observed at about 1700 cm^−1^ [36]. -CH bending bonds of CH_2_ were observed around 1470 cm^−1^, while C-O stretching bonds of PVP were observed between 1250 and 1200 cm^−1^. The spectra of the annealed fibers showed the presence of some functional groups. This confirmed that annealing in an inert environment did not fully decompose the polymer. The peak around 2300 cm^−1^ can be caused by C-N stretching movements [37]. Peaks due to stretching vibrations of O-W bonds were observed at about 600 cm^−1^.

**Figure 7:**
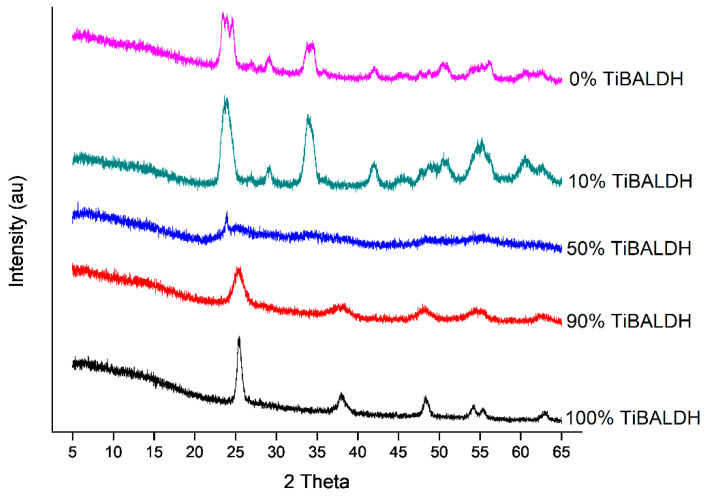
XRD patterns of the fibers after annealing.

The XRD patterns of the fibers after heat treatment in argon are shown in Figure 7. Fibers containing 100% TiBALDH had diffraction peaks characteristic of anatase TiO_2_ at 25.5°, 39.0°, 48.1°, 54.0°, and 63.0° assigned to (101), (112), (200), (105), and (204) planes, respectively [38,39]. Moreover, 0% TIBALDH had peaks typical of monoclinic WO_3_ at 23.1°, 23.5°, 24.4°, and 33.4° corresponding to (002), (020), (200), and (022) planes, respectively [34]. Fibers containing TiBALDH and AMT were less crystalline. Further, 90% TiBALDH and 10% TiBALDH had peaks for anatase TiO_2_ and monoclinic WO_3_.

**Figure 8:**
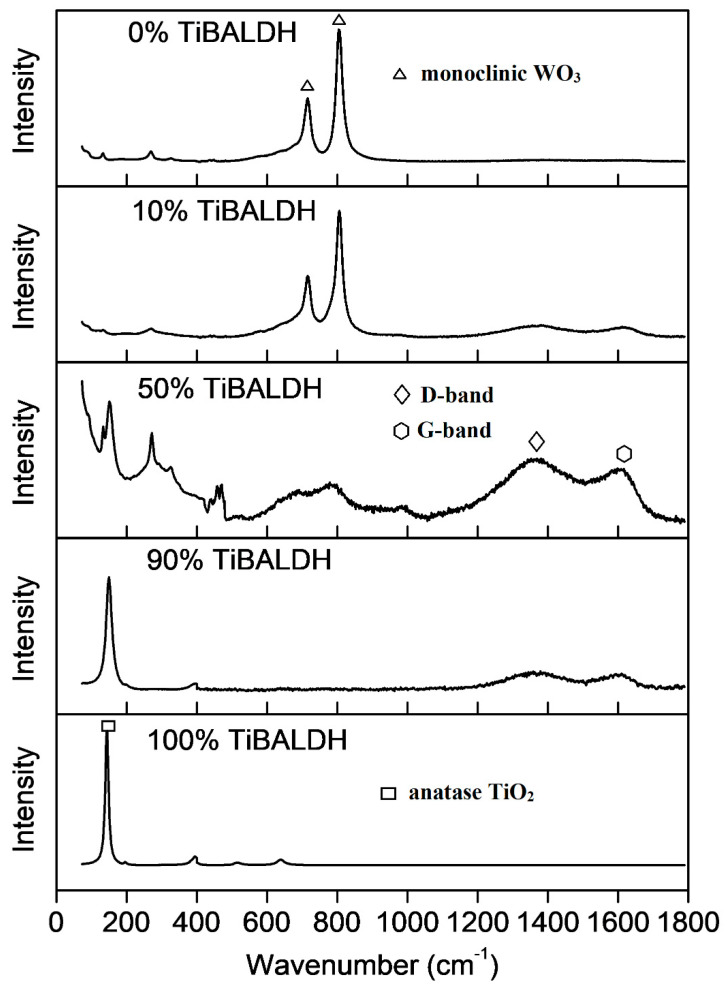
Raman spectra of the fibers after annealing.

Raman spectra obtained after the fibers were annealed is shown in Figure 8. Fibers prepared from 100% TiBALDH had peaks at 144, 399, 515, and 630 cm^−1^ characteristic of anatase TiO_2_ [40]. For 0% TiBALDH, peaks around 250–330 cm^−1^ associated with O-W-O bending vibrations were not observed because the fibers were not highly crystalline. However, the intense peaks around 710 and 801 cm^−1^ are Raman peaks for monoclinic WO_3_, which can be attributed to the O-W-O stretching vibrations [41]. Fibers containing both TiBALDH and AMT showed peaks associated with anatase TiO_2_ and monoclinic WO_3_ and broad peaks at 1350 and 1600 cm^−1^, assigned to D and G bands carbon, respectively [42].

**Figure 9:**
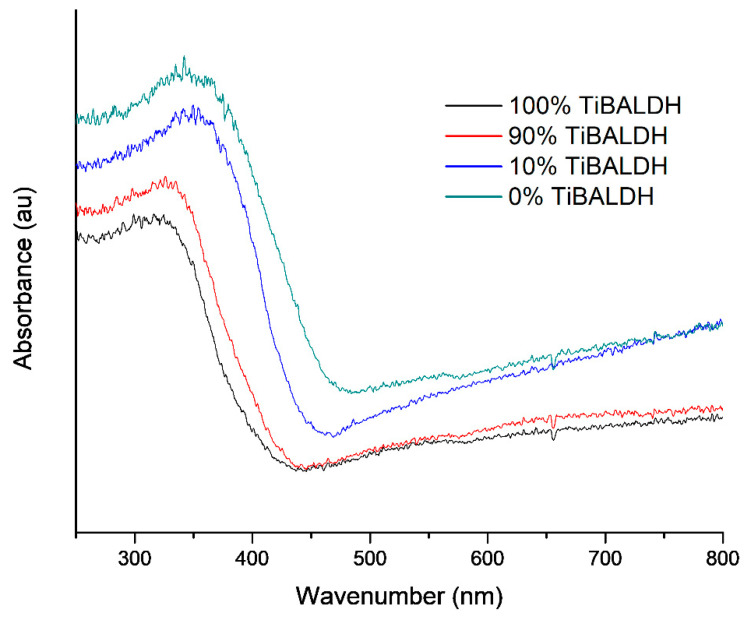
UV–VIS diffuse reflectance spectra of samples after annealing.

Figure 9 presents the diffuse reflectance UV–VIS spectra. The absorption edge of the composite fibers increased to the visible light region of the spectrum. C and nitrogen atoms in TiO_2_/WO_3_ fibers allow valence electrons in the TiO_2_ band gap to be excited at a wavelength greater than 370 nm. The increase in the excitation wavelength corresponded to the decrease in the amount of TiO_2_ precursor. The shift in absorption edge is confirmed by the band gap of the fibers shown in Table 2. The indirect band gaps were calculated by extrapolating the linear portions of Tauc plots based on the Kubelka-Munk function. The Tau plots are shown in Figure S1. The reduction in band gap energy shows that the fibers can absorb light visible light, improving their photocatalytic efficiency. [43]. Fibers prepared from 50% TiBALDH were black and, therefore, absorbed light and did not have reflectance spectra.

**Figure 10:**
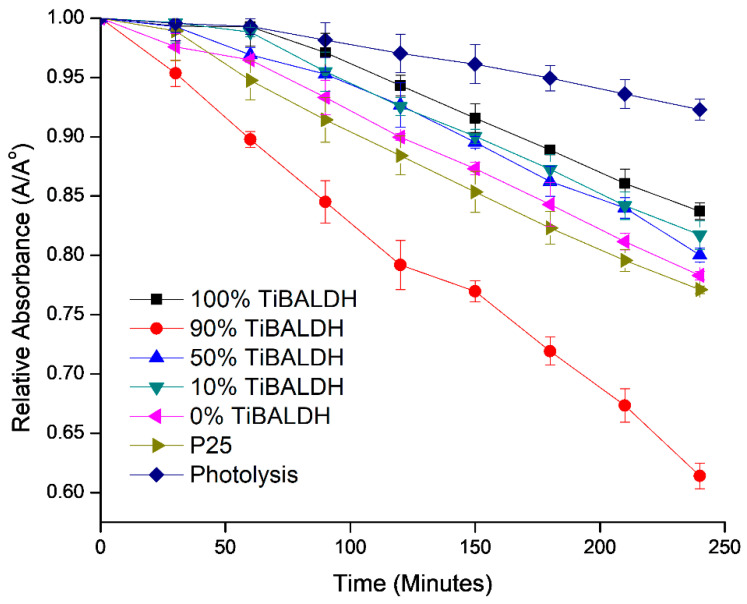
Photocatalytic activity of annealed fibers on methylene blue in visible light.

Figure 10 shows the photocatalytic degradation of methylene blue by the fibers in visible light. The set up for photocatalysis is shown in Figure S2 while the absorbance values recorded during the photocatalysis process are shown in Table S1. All the fibers demonstrated photocatalytic activity in visible light. Fibers with 100% TiBALDH had the least photocatalytic effect in visible light. Fibers prepared from 90% TiBALDH had the greatest degradation effect on methylene blue dye after 4 h, with a photocatalytic activity about two times better than P25 TiO_2_. The photocatalytic activity was also comparable to the performance of other N-containing, TiO_2_ photocatalysts. Choi et al. reported 80% degradation of methylene blue in visible light by TiO_2_/WO_3_-based films doped with nitrogen [26]. WO_3_-N-TiO_2_ nanosheets synthesized by Lee et al. degraded 43.4% of hexane vapor in visible light [27]. Combining TiO_2_ with WO_3_ and C and N allows absorption of visible light due to additional allowed energy levels in the band gap of TiO_2_, which decreases the rate of electron hole recombination during photocatalysis. This improves the photocatalytic activity of TiO_2_. A graph of −ln(A/A^o^) against time, shown in Figure S3, was used to determine the rate constants for the photocatalysis processes. The results are shown in Table 3. Fibers that showed a higher degradation rate of methylene blue had larger K*_app_* values.

## 4. Conclusions

TiO_2_/WO_3_/C/N composite nanofibers were prepared by electrospinning followed by annealing in argon. The polymer component of the fiber pyrolyzes during the annealing process to form a residue made of carbon and nitrogen. Characterization by XPS, SEM-EDX, FTIR, XRD, and Raman spectroscopy showed that C, N, Ti, and W were present in the composite fibers in different proportions. The UV–VIS DRS showed that the absorbed light at a higher wavelength than the values reported for pure TiO_2_ fibers. The decrease in the fibers’ band gaps showed that the photocatalytic activity of TiO_2_ could be improved by coupling TiO_2_ with WO_3_ and nonmetals such as carbon and nitrogen. The fiber’s C/N phase can sensitize the system to visible light and enhance the charge separation during photocatalysis. The degradation of methylene blue by the annealed fibers in visible light was studied. Fibers containing 90% TiBALDH had the highest C and N content, and they showed the most significant photocatalytic activity.

## Figures and Tables

**Table 1:** Elemental composition of the annealed fibers based on XPS and energy-dispersive X-ray (EDX).

Sample		C	O	Ti	W	N
	At%					
100% TiBALDH	XPS	35.3	52.4	10.3		2.0
	EDX	30.1	47.2	18.2		4.5
90% TiBALDH	XPS	24.9	57.6	11.7	3.5	2.3
	EDX	40.4	44.2	6.3	1.0	8.1
50% TiBALDH	XPS	50.7	38.0	1.5	4.8	5.0
	EDX	22.1	51.8	9.6	14.5	2.0
10% TiBALDH	XPS	28.7	54.7	2.8	12.6	1.2
	EDX	21.1	41.8	1.3	34.7	1.1
0% TiBALDH	XPS	30.0	52.5		15.9	1.6
	EDX	14.2	42.5		41.7	1.6

**Table 2:** Band gap values for annealed fibers.

Fibers	100% TiBALDH	90% TiBALDH	10% TiBALDH	0% TiBALDH
Band gap (eV)	2.9	2.7	2.6	2.4

**Table 3:** Values of rate constant and *r*^2^ for the photocatalysis decomposition of methylene blue under visible light.

Sample	K*_app_* (min^−1^)	*r* ^2^
100% TiBALDH	0.0009	97.8
90% TiBALDH	0.002	99.0
50% TiBALDH	0.001	98.5
10% TiBALDH	0.001	99.2
0% TiBALDH	0.0011	99.2
P25	0.0012	99.9
Bare methylene blue	0.0004	98.5

## Data Availability

The study did not report any data.

## Supplementary Materials

The following are available online at https://www.mdpi.com/2079-4991/11/2/351/s1, Figure S1: Tauc plots for fibers: (a) 100% TiBALDH (b) 90% TiBALDH, (c) 10% TiBALDH, and (d) 0% TiBALDH; Figure S2: Photocatalysis setup: a) fibers and dye allowed to stand overnight and b) mixture in visible light before absorption measurements are done using a Jasco V-550 UV–VIS spectrometer; Figure S3: Apparent rate constant and r^2^ values for the photocatalytic degradation of methylene blue in visible light; Table S1. Absorbance values for the photocatalytic process of methylene blue degradation in visible light.
