# Supplementary material for: Preparation of TiO2/WO3/C/N Composite Nanofibers by Electrospinning Using Precursors Soluble in Water and Their Photocatalytic Activity in Visible Light
Source: Nanomaterials (Basel). 2021 Feb 1;11(2):351. doi: 10.3390/nano11020351 (PMC7912636; doi:10.3390/nano11020351)
Supplement: Supplementary file 1 [file nanomaterials-11-00351-s001.pdf]

Supplementary information

Preparation of TiO_2/WO_3/C/N Composite Nanofibers by Electrospinning Using Precursors Soluble in Water and Their Photocatalytic Activity in Visible light

Vincent Otieno Odhiambo [1*], Chra Rasool [1], Le Ba Thong [1], Zoltán Kónya[2], Csaba Cserháti[3], Zoltán Erdélyi[3], István Endre Lukács[4], Imre Miklós Szilágyi[1]

[1]Budapest University of Technology and Economics, Department of Inorganic and Analytical Chemistry, H-1111 Budapest, Szent Gellért tér 4., Hungary

[2]University of Szeged, Department of Applied and Environmental Chemistry, H-6720 Szeged, Rerrich Béla tér 1., Hungary

[3]University of Debrecen, Faculty of Sciences and Technology, Department of Solid State Physics, H-4026 Debrecen, Bem ter 18/b, Hungary

[4]Research Institute for Technical Physics and Materials Science, Hungarian Academy of Sciences, Konkoly Thege M. út 29-33., Budapest, H-1121, Hungary

Correspondence: vincent.odhiambo@mail.bme.hu and imre.szilagyi@mail.bme.hu

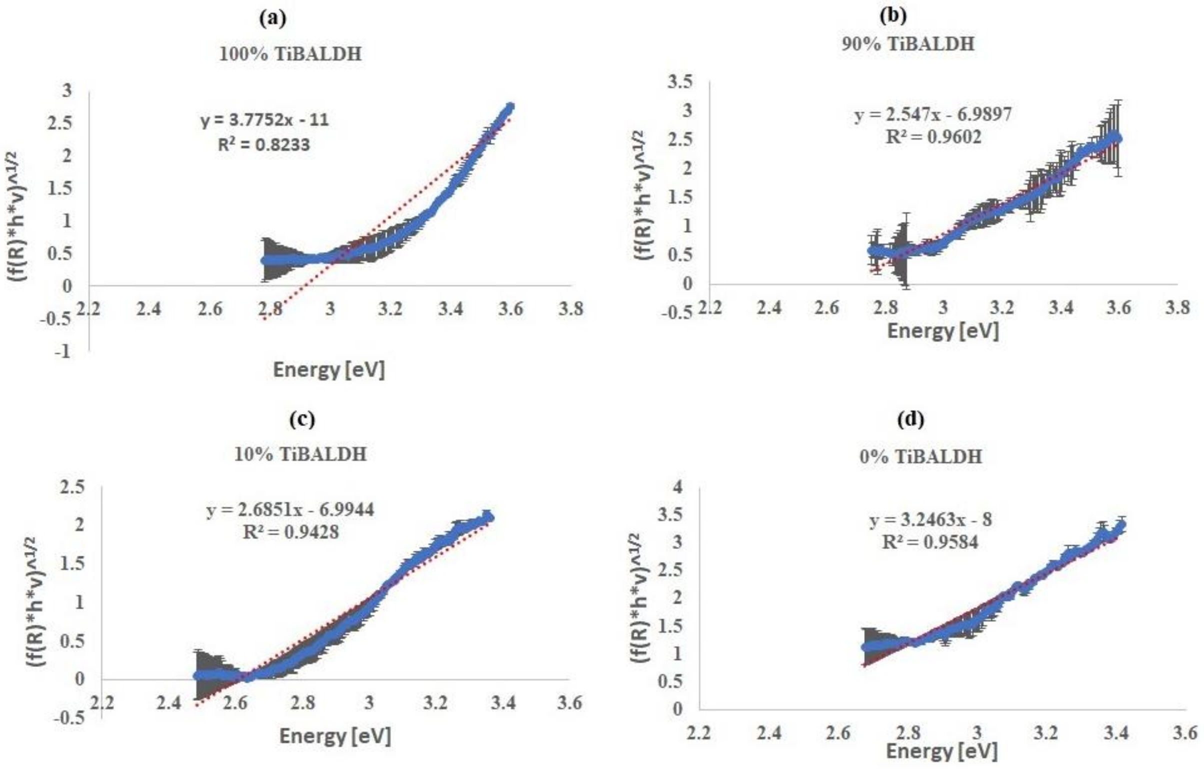

Figure S1. Tauc plots a) 100% TiBALDH b) 90% TiBALDH c) 10% TiBALDH and d) 0% TiBALDH

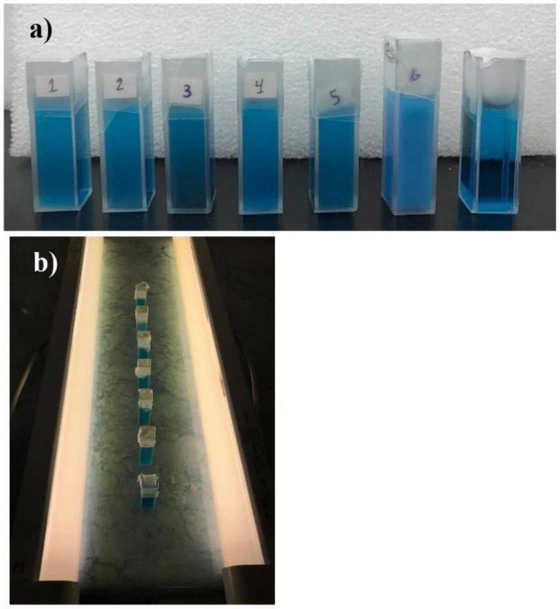

Figure S2. Photocatalysis set up **a**) Fibers and dye allowed to stand overnight **b**) mixture in visible light before absorption measurements are done using a Jasco V-550 UV-VIS spectrometer

Table S1. Absorbance values for photocatalytic process of methylene blue degradation in visible light

	Absorbance						
Time (Min)	100% TiBALDH	90% TiBALDH	50% TiBALDH	10% TiBALDH	0% TiBALDH	P25	Bare methylene blue
0	2.31656	2.31042	2.29842	2.31987	2.01493	2.31236	2.32121
30	2.30082	2.203075	2.28195	2.311089	1.96609	2.287553	2.36612
60	2.29987	2.074053	2.227594	2.292482	1.94385	2.191027	2.34984
90	2.24933	1.952477	2.189794	2.215253	1.88041	2.113883	2.32266
120	2.18495	1.829468	2.13021	2.147228	1.81292	2.044561	2.29596
150	2.12146	1.778218	2.058037	2.088607	1.75917	1.973696	2.2745
180	2.05925	1.661693	1.982109	2.02423	1.69845	1.903348	2.24647
210	1.99392	1.555856	1.93076	1.953409	1.63524	1.840046	2.21482
240	1.93938	1.399673	1.877772	1.895914	1.57763	1.78283	2.18377

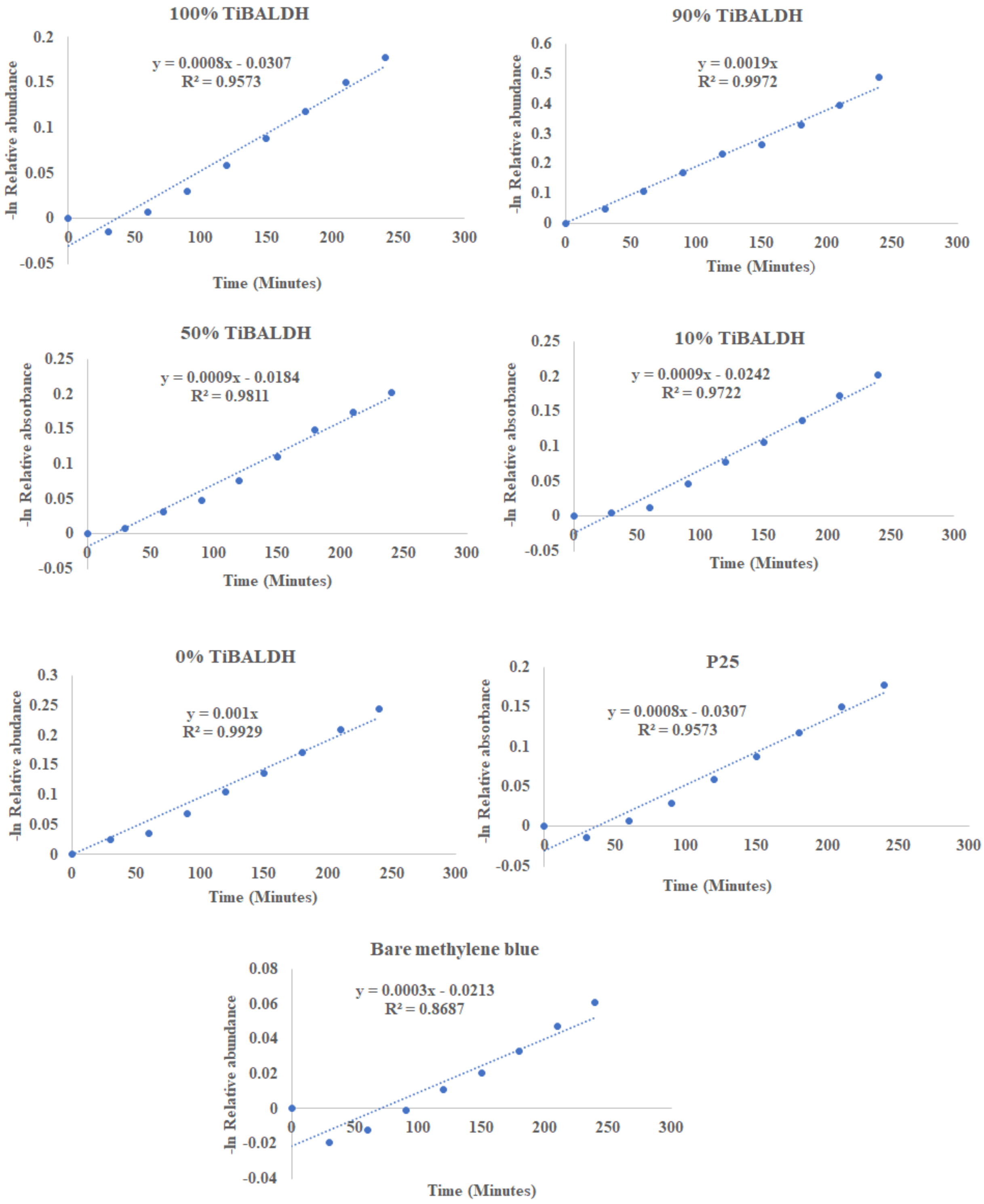

Figure S3. Apparent rate constant and R^2 values for the photocatalytic degradation of methylene blue in visible light
